# Plasma Phospholipid Fatty Acid Concentration and Incident Coronary Heart Disease in Men and Women: The EPIC-Norfolk Prospective Study

**DOI:** 10.1371/journal.pmed.1001255

**Published:** 2012-07-03

**Authors:** Kay-Tee Khaw, Marlin D. Friesen, Elio Riboli, Robert Luben, Nicholas Wareham

**Affiliations:** 1 Department of Public Health and Primary Care, University of Cambridge School of Clinical Medicine, Cambridge, United Kingdom; 2 Department of Environmental Health Sciences, Johns Hopkins Bloomberg School of Public Health Baltimore, Maryland, United States of America; 3 School of Public Health, Imperial College, London, United Kingdom; 4 MRC Epidemiology Unit, Cambridge, United Kingdom; Vrije Universiteit Amsterdam, Netherlands

## Abstract

**Background:**

The lack of association found in several cohort studies between dietary saturated fat and coronary heart disease (CHD) risk has renewed debate over the link between dietary fats and CHD.

**Methods and Findings:**

We assessed the relationship between plasma phospholipid fatty acid (PFA) concentration and incident CHD using a nested case control design within a prospective study (EPIC-Norfolk) of 25,639 individuals aged 40–79 years examined in 1993–1997 and followed up to 2009. Plasma PFA concentrations were measured by gas chromatography in baseline samples retrieved from frozen storage. In 2,424 men and women with incident CHD compared with 4,930 controls alive and free of cardiovascular disease, mean follow-up 13 years, saturated PFA (14:0, 16:0,18:0) plasma concentrations were significantly associated with increased CHD risk (odds ratio [OR] 1.75, 95% CI 1.27–2.41, *p<*0.0001), in top compared to bottom quartiles (Q), and omega-6 polyunsaturated PFA concentrations were inversely related (OR 0.77, 0.60–0.99, *p<*0.05) after adjusting for age, sex, body mass index, blood pressure, smoking, alcohol intake, plasma vitamin C, social class, education, and other PFAs. Monounsaturated PFA, omega-3 PFA, and trans PFA concentrations were not significantly associated with CHD. Odd chain PFA (15:0, 17:0) concentrations were significantly inversely associated with CHD (OR 0.73, 0.59–0.91, *p<*0.001, Q4 versus Q1). Within families of saturated PFA or polyunsaturated PFA, significantly heterogeneous relationships with CHD were observed for individual fatty acids.

**Conclusions:**

In this study, plasma concentrations of even chain saturated PFA were found to be positively and omega-6 polyunsaturated PFA inversely related to subsequent coronary heart disease risk. These findings are consistent with accumulating evidence suggesting a protective role of omega-6 fats substituting for saturated fats for CHD prevention.

*Please see later in the article for the Editors' Summary*

## Abstract 2

Kay-Tee Khaw and colleagues analyze data from a prospective cohort study and show associations between plasma concentrations of saturated phospholipid fatty acids and risk of coronary heart disease, and an inverse association between omega-6 polyunsaturated phospholipid fatty acids and risk of coronary heart disease.

## Editors' Summary

**Background:**

Coronary heart disease (CHD) is a condition caused by a build-up of fatty deposits on the inner walls of the blood vessels that supply the heart, causing the affected person to experience pain, usually on exertion (angina). A complete occlusion of the vessel by deposits causes a heart attack (myocardial infarction). Lifestyle factors, such as diet (particularly one high in fat), contribute to causing CHD. There are different types of fat, some of which are thought to increase risk of CHD, such as saturated fat, typically found in meat and dairy foods. However, others, such as unsaturated fats (polyunsaturated and monounsaturated fats) found in foods such as vegetable oils, fish, and nuts, may actually help prevent this condition.

**Why Was This Study Done?:**

Although there have been many studies investigating the role of different types of dietary fat in coronary heart disease, it is still not clear whether coronary heart disease can be prevented by changing the type of dietary fat consumed from saturated to unsaturated fats or by lowering all types of dietary fat. Furthermore, many of these studies have relied on participants recalling their dietary intake in questionnaires, which is an unreliable method for different fats. So in this study, the researchers used an established UK cohort to measure the levels of different types of fatty acids in blood to investigate whether a diet high in saturated fatty acids and low in unsaturated fatty acids increases CHD risk.

**What Did the Researchers Do and Find?:**

The researchers used a selection of 10,000 participants (all men and women aged 40–79 years) from the prospective European Prospective Investigation into Cancer (EPIC)-Norfolk cohort. Blood samples from the selected participants taken at the start of the study in 1993–1997 were analyzed to determine levels of specific fatty acids. Participants were followed up till 2011. The researchers identified 2,424 participants who were subsequently diagnosed with CHD using death certificates and hospital discharge data and matched these with 4,930 controls who were still alive and free of known coronary disease. The researchers grouped the type of blood fatty acids identified in the blood samples into six families (even chain saturated fatty acid, odd chain saturated fatty acid, omega-6 polyunsaturated fatty acid, omega-3 polyunsaturated fatty acid, monounsaturated fatty acid, and trans-fatty acid), which represented saturated and unsaturated fatty acids. Using statistical methods, the researchers then compared the risks of developing CHD between cases and controls by the concentration of fatty acid families after adjusting for age and sex and other factors, such as body mass index, physical activity, and smoking. Using these methods, the researchers found that there was no overall significant relationship between total blood fatty acid concentration and CHD but there was a positive association with increasing blood saturated fatty acid concentration after adjusting for other fatty acid concentrations, with an odds ratio of 1.83 comparing higher versus lower concentrations. This risk was attenuated after adjusting for cholesterol levels, indicating that much of the association between saturated fatty acid and CHD is likely to be mediated through blood cholesterol levels. In contrast, blood omega-6 poly-unsaturated fatty acid concentrations were associated with lower CHD risk. Blood monounsaturated fatty acids, omega-3 poly-unsaturated fatty acids, and trans-fatty acids were not consistently associated with CHD risk. The authors also noted that within families of fatty acids, individual fatty acids related differently to CHD risk.

**What Do These Findings Mean?:**

These findings suggest that plasma concentrations of saturated fatty acids are associated with increased risk of CHD and that concentrations of omega-6 poly-unsaturated fatty acids are associated with decreased risk of CHD. These findings are consistent with other studies and with current dietary advice for preventing CHD, which encourages substituting foods high in saturated fat with n-6 polyunsaturated fats. The results also suggest that different fatty acids may relate differently to CHD risk and that the overall balance between different fatty acids is important. However, there are limitations to this study, such as that factors other than diet (genetic differences in metabolism, for example) may cause changes to blood fatty acid levels so a major question is to identify what factors influence blood fatty acid concentrations. Nevertheless, these findings suggest that individual fatty acids play a role in increasing or decreasing risks of CHD.

**Additional Information:**

Please access these Web sites via the online version of this summary at http://dx.doi.org/10.1371/journal.pmed.1001255.

- Information about the EPIC-Norfolk study is available
- The American Heart Foundation provides patient-friendly information about different dietary fats as does Medline
- The British Heart Foundation also provides patient-friendly information on heart conditions

## Introduction

Dietary fat intake has long been implicated in the aetiology of coronary heart disease (CHD). The adverse relationship of transfats with CHD is widely accepted [1] such that recommendations and legislation controlling use of transfats are widespread.

A meta-analysis of prospective cohort studies concluding that there is no significant evidence that dietary saturated fat is associated with increased risk of cardiovascular disease has renewed debate over fat and CHD [2],[3]. Randomized trials are not always conclusive. The Women's Health Initiative (WHI) found no difference in cardiovascular outcomes in women randomized to a low fat diet compared to controls [4]. However, though the intervention group lowered total fat intake, the polyunsaturated fat/saturated fat ratio in the WHI trial was unchanged. It is notable that trials reporting significant differences in cardiovascular outcomes altered dietary fatty acid (FA) composition such as unsaturated/saturated fat ratios rather than simply lowering total fat intakes [5]–[7].

Observational studies using self-reported dietary instruments have limitations accurately assessing intake of different fats. Blood FA profiling may provide a more objective and accurate biomarker of FA intake [8]–[13]. Studies to date examining the prospective relationship between blood FA concentrations and CHD have had limited numbers of events, and few reported the whole range of FAs [14]–[23].

We tested the hypothesis that high saturated FA and low polyunsaturated plasma phospholipid fatty acid (PFA) status increase CHD risk in a prospective population study and explored the relationship of individual FAs with CHD.

## Methods

The European Prospective Investigation into Cancer (EPIC)-Norfolk is a prospective study of 25,639 men and women aged 40–79 years in Norfolk, UK similar in characteristics to UK general population samples, who participated in a baseline survey in 1993–1997 [24]. Participants completed a health and lifestyle questionnaire including data on medical history, smoking, alcohol intake, physical activity, social class, and education [25] and attended a clinic for a health examination. Body mass index (BMI) was calculated as weight (kilograms) divided by height (metres) squared. Blood pressure was measured using an Accutorr non-invasive blood pressure monitor. Blood samples were spun, separated into 0.5 ml fractions of serum and citrated plasma, placed in straws, sealed, and stored in liquid nitrogen. Fresh samples were assayed for vitamin C and lipids [26].

### Measurement of Plasma Phospholipid FA Concentrations

Funding was obtained for blood FA analyses in 2003–2008. Selection of participants for analyses was based on a series of nested case control studies with incident cases of cancers and cardiovascular disease and up to four disease-free controls for each case. This selection totalled about 10,000 individuals representing two-fifths of the cohort, enriched for incident diseases. Analyses on 8,000 samples were carried out in the WHO International Agency for Cancer Research laboratories, Lyon, France [27]. Because of laboratory constraints in Lyon, an additional 2,000 samples were analysed in Quotient Laboratories, UK, using the same methods and quality control standards.

Citrated plasma straws retrieved from storage were thawed, di-palmitoyl-D_31_-phosphatidylcholine Sigma) internal standard was added to each plasma sample, total lipids extracted, and purified by adsorption chromatography (LC-Si SPE, Supelco/Sigma). Plasma phospholipids were analysed by gas chromatography. Concentrations were measured by comparison of peak areas of individual FAs with the peak area of the internal standard using individual calibration curves. The method allowed for the analysis of individual FAs belonging to six classes of FAs. We analysed data for 22 FAs: saturated even chain fatty acid (SFA) (14:0, 16:0, 18:0); odd chain FA (15:0, 17:0); omega-6 polyunsaturated, n-6 polyunsaturated fatty acid (PUFA) (18:2n-6, 18:3n-6, 20:2n-6, 20:3n-6, 20:4n-6, 22:4n-6, and 22:5n-6); omega-3 polyunsaturated, n-3 PUFA (18:3n-3, 20:5n-3, 22:5n-3, 22:6n-3); mono-unsaturated fatty acid (MUFA) (16:1n-7, 18:1n-7cis, 18:1n-9cis, 20:1n-9); and trans-fatty acid (16:1n9trans, 18:1n9trans). The methods are detailed in Text S1.

### Ascertainment of Coronary Heart Disease Events

All participants are flagged for death certification with the National Health Service (NHS) Central Register, UK with death certificates coded by nosologists according to the International Classification of Disease (ICD). CHD death was defined using ICD9 410–414 or ICD10 I22–I25 as underlying cause of death. Data linkage with the East Norfolk Health Authority database identifies all hospital contacts for participants using their NHS number. We used the ICD diagnostic codes listed to ascertain hospital episodes for CHD. Participants were identified as having a CHD event during follow-up if they had a hospital admission and/or died with CHD as cause of death, with clinical validation through medical record inspection of a sample [28]. We ascertained fatal and non-fatal CHD events to December 2009.

### Selection of Cases and Controls

From all the individuals with available measurement of plasma phospholipid FAs, we identified 2,424 individuals who had incident CHD and 4,930 control individuals alive and free of known cardiovascular disease during follow-up to 2009.

### Data Analysis

FAs were grouped into six families a priori: even chain SFA, odd chain FA, n-6 PUFA, n-3 PUFA, MUFA, and trans-fatty acid. Analyses used SPSS Version 17.0. We compared baseline characteristics in individuals with incident CHD and controls. We examined the odds ratios for CHD by quartile of concentration of FA families adjusting first for age and sex, then in multivariate models adjusting for all the other FA families, then additionally adjusting for covariates: BMI, physical activity, cigarette smoking habit, alcohol intake, social class, education, plasma vitamin C (used as a biomarker of fruit and vegetable intake [26]), personal history of diabetes, and systolic blood pressure then finally additionally adjusting for blood cholesterol. We repeated the analyses using FAs categorised using mol%

We then examined the odds ratios for CHD with individual FAs. In these models we used each individual FA as a continuous variable, adjusting for all the other individual acids as continuous variables. To enable comparisons, the interval used to estimate the odds ratio was approximately one standard deviation (SD) for each FA. All models were age and sex adjusted.

Trans-fatty acids were at very low concentrations near the lower limit of determination. Quality controlled results were only available from the laboratory for 1,979 cases and 3,995 controls. We analysed trans-fatty acid data two ways: coding missing data as missing or as zero on the basis that these were extremely low values. Since findings were similar using either approach, we present results for the more conservative approach of analysing the data as missing.

## Results

Table 1 shows baseline characteristics of the 2,434 individuals with incident CHD (633 fatal) and 4,930 controls. Those who developed CHD compared to controls were older, had higher blood cholesterol, low-density lipoprotein (LDL) cholesterol, triglycerides, and blood pressure levels and lower high-density lipoprotein (HDL) cholesterol. They were more likely to have diabetes, be smokers and physically inactive, and have lower plasma vitamin C concentrations and lower mean alcohol intake. Quantitatively, the most abundant phospholipid FAs were palmitic (16:0), linoleic (18:2n-6), stearic (18:0), and arachidonic acid (AA) (20:4n-6). Plasma concentrations of phospholipid trans-fatty acid contributed less than 0.2% of the total.

**Table 1:** Baseline characteristics of men and women with incident CHD and controls EPIC-Norfolk 1993–2009.

Characteristics	CHD Cases	Controls	*p*-Value
M**en**	*n = *1,595	*n = *2,246	
**Mean (SD)**			
Age (y)	64.9 (7.6)	60.0 (8.0)	<0.001
BMI (kg/m^2^)	27.2 (3.4)	26.3 (3.1)	<0.001
Systolic blood pressure (mmHg)	143.1 (18.8)	136.1 (16.4)	<0.001
Diastolic blood pressure (mmHg)	85.7 (12.0)	83.7 (10.6)	<0.001
Total cholesterol (mmol/l)	6.25 (1.13)	6.03 (1.05)	<0.001
LDL-cholesterol (mmol/l)	4.11 (0.98)	3.92 (0.95)	<0.001
HDL-cholesterol (mmol/l)	1.17 (0.31)	1.25 (0.33)	<0.001
Triglycerides (mmol/l)	2.22 (1.23)	2.01 (1.15)	<0.001
Plasma vitamin C (µmol/l)	43.6 (18.9)	48.6 (18.2)	<0.001
Alcohol (units/week)	8.65 (11.4)	9.74 (11.1)	0.006
PFA			
Total PFA (µmol/l)	4,721.9 (1215.6)	4,514.2(1196.8)	0.007
Saturated PFA (µmol/l)	1,852.8 (483.3)	1,798.0(460.5)	<0.001
Odd chain PFA (µmol/l)	27.6 (8.1)	27.2 (7.9)	0.16
Omega-6 polyunsaturated PFA (µmol/l)	1,820.5 (513.3)	1,810.0 (495.0)	0.52
Omega-3 polyunsaturated PFA (µmol/l)	362.9 (161.5)	347.7 (362.9)	0.003
Monounsaturated PFA (µmol/l)	558.2 (222.5)	531.2 (207.1)	<0.001
Trans PFA (µmol/l)	7.0 (4.1)	6.8 (4.1)	0.09
Saturated PFA (% total)	40.2 (2.7)	40.0 (2.8)	0.01
Odd chain PFA (% total)	0.6 (0.1)	0.6 (0.1)	0.05
Omega-6 polyunsaturated PFA (% total)	39.5 (3.7)	40.2(3.6)	<0.001
Omega-3 polyunsaturated PFA (% total)	7.8 (2.5)	7.6 (2.3)	0.01
Monounsaturated PFA (% total)	11.8(2.3)	11.5 (2.1)	<0.001
Trans PFA (% total)	0.1 (0.1)	0.1 (0.1)	0.66
**Percent (** ***n*** **)**			
History of diabetes	7.3 (116)	2.1 (48)	<0.001
Smoking status			
Never	23.7 (375)	36.1 (804)	<0.001
Former	62.9 (995)	54.8 (1221)	
Current	13.3 (211)	9.1 (203)	
Physical activity			
Inactive	38.7 (617)	25.5 (572)	<0.001
Moderately inactive	24.5 (391)	25.8 (580)	
Moderately active	20.7 (330)	24.3 (546)	
Active	16.0 (265)	24.3 (547)	
Education level			
No qualifications	38.5 (614)	29.4 (659)	<0.001
Junior school	7.1 (113)	8.3 (187)	
Senior school	44.4 (707)	44.9 (1007)	
Diploma or degree	10.0(159)	17.4 (391)	
Social class			
Non-manual	56.5 (874)	60.4 (1339)	0.01
Manual	43.5 (672)	39.6 (878)	
**Women**			
**Mean (SD)**	*n* = 776	*n = *2,684	
Age (y)	66.5 (7.1)	59.4 (8.5)	<0.001
BMI (kg/m^2^)	27.3 (4.5)	25.9 (3.9)	<0.001
Systolic blood pressure (mmHg)	142.1 (19.7)	132.6 (18.0)	<0.001
Diastolic blood pressure (mmHg)	80.9 (11.1)	80.0 (10.7)	<0.001
Total cholesterol mmol/l	6.85 (1.28)	6.35 (1.20)	<0.001
LDL-cholesterol mmol/l	4.47 (1.13)	4.03 (1.06)	<0.001
HDL-cholesterol mmol/l	1.45 (0.40)	1.58 (0.42)	<0.001
Triglycerides mmol/l	2.10 (1.15)	1.64 (1.07)	<0.001
Plasma vitamin C (µmol/l)	53.1 (20.2)	59.8 (19.9)	<0.001
Alcohol (units/week)	3.1 (4.6)	4.5 (5.7)	<0.001
PFA			
Total PFA (µmol/l)	5,066.0 (1333.7)	4,975.1 (1,261.8)	0.07
Saturated PFA (µmol/l)	2,048.1 (530.0)	1,980.8 (487.9)	<0.001
Odd chain PFA (µmol/l)	30.9 (9.1)	30.9 (8.5)	0.96
Omega-6 polyunsaturated PFA (µmol/l)	1,976.6 (544.0)	1,968.3 (528.6)	0.69
Omega-3 polyunsaturated PFA (µmol/l)	402.1 (173.1)	402.2 (170.7)	0.98
Monounsaturated PFA (µmol/l)	608.4 (228.8)	593.1 (203.6)	0.06
Trans PFA (µmol/l)	7.9 (4.5)	7.5(4.0)	0.02
Saturated PFA (% total)	40.5 (2.5)	39.9 (2.7)	<0.001
Odd chain PFA (% total)	0.6 (0.1)	0.6 (0.1)	0.34
Omega-6 polyunsaturated PFA (% total)	39.1 (3.5)	39.6 (3.5)	<0.001
Omega-3 polyunsaturated PFA (% total)	7.9 (2.3)	8.0 (2.4)	0.20
Monounsaturated PFA (% total)	11.8 (2.2)	11.7 (1.9)	0.40
Trans PFA (% total)	0.1 (0.1)	0.1 (0.1)	0.11
**Percent (** ***n*** **)**			
History of diabetes	5.5 (46)	1.3 (36)	<0.001
Smoking status			
Never	50.5 (417)	60.0 (1599)	<0.001
Former	36.6 (302)	31.2 (833)	
Current	12.8 (106)	8.8 (235)	
Physical activity			
Inactive	45.2 (379)	25.5 (685)	<0.001
Moderately inactive	30.2 (253)	33.9 (909)	
Moderately active	14.8 (124)	24.3 (652)	
Active	9.8 (82)	16.3 (437)	
Education level			
No qualifications	63.2 (530)	47.9 (1284)	<0.001
Junior school	10.5 (88)	16.1 (433)	
Senior school	22.2 (186)	25.2 (677)	
Diploma or degree	4.1 (34)	10.8 (289)	
Social class			
Non-manual	59.1 (476)	63.8 (1676)	0.008
Manual	40.9 (330)	36.2 (949)	

Table 2 shows characteristics of the participants by quartile (Q) of plasma phospholipid SFA. Plasma phospholipid SFA concentrations correlated with the other FA concentrations as well as age, blood pressure, and lipids. A positive relationship was seen with alcohol intake, but no consistent relationships with smoking habit, physical activity, diabetes history, and social class.

**Table 2:** Baseline characteristics of participants by quartile of plasma saturated PFA concentration.

Characteristics	Quartile of Plasma-Saturated PFA Concentration				
	1	2	3	4	*p*-Value*
**Men**					
**Mean (SD)**					
Age (y)	61.4 (6.2)	62.1 (8.1)	62.3 (8.0)	62.6 (8.1)	0.014
BMI (kg/m^2^)	26.2 (3.2)	26.6 (3.2)	26.9 (3.0)	27.3 (3.4)	<0.0001
Systolic blood pressure (mmHg)	136.3 (17.1)	138.6 (17.9)	140.2 (17.8)	142.7 (17.6)	<0.0001
Total cholesterol (mmol/l)	5.54 (0.91)	6.01 (0.96)	6.43 (1.01)	6.82 (1.1)	<0.0001
LDL cholesterol (mmol/)	3.66 (0.86)	4.0 (0.92)	4.2 (0.96)	4.4 (1.01)	<0.0001
HDL cholesterol (mmol/l)	1.2 (0.30)	1.21 (0.32)	1.24 (0.35)	1.23 (0.34)	0.018
Triglyceride (mmol/l)	1.52 (0.69)	1.94 (0.85)	2.28 (1.03)	3.04 (1.71)	<0.0001
Plasma vitamin C (µmol/l)	46.6 (18.6)	45.6 (19.0)	48.3 (18.4)	45.5 (18.4)	0.004
Alcohol (units/wk)	7.4 (9.1)	8.9 (10.9)	9.9 (11.1)	12 (14.1)	<0.0001
Plasma PFA total (µmol/l)	3,412 (456)	4,253 (362)	4,991 (460)	6,384 (1,077)	<0.0001
Saturated PFA (µmol/l)	1,354 (163)	1,701 (79)	1,993 (99)	2,562 (375)	<0.0001
Odd chain PFA (µmol/l)	21.9 (4.6)	26.1 (5.1)	29.5 (6.3)	35.8 (9.3)	<0.0001
Omega-6 PFA (µmol/l)	1,399 (244)	1,709 (257)	1,974 (304)	2,466 (524)	<0.0001
Omega-3 PFA(µmol/l)	259 (97)	329 (110)	395 (138)	498 (198)	<0.0001
Monounsaturated PFA (µmol/l)	379 (86)	489 (95)	599 (146)	823 (245)	<0.0001
Trans PFA (µmol/l)	5.6 (3.4)	6.6 (3.5)	7.2 (3.9)	8.9 (5.1)	<0.0001
**Percent (** ***n*** **)**					
History of diabetes	4.5 (55)	3.7 (37)	3.8 (34)	5.3 (38)	0.35
Current smoker	12.3 (149)	10.0 (99)	9.4 (84)	1.5 (82)	0.14
Physically inactive	30.7 (374)	30.7 (307)	30.7 (275)	32.3 (233)	0.70
No educational qualifications	33.3 (406)	33.1 (331)	33.3 (299)	32.9 (237)	0.79
Manual social class	42.7 (510)	40.9 (402)	50.6 (356)	39.8 (282)	0.61
**Women**					
**Mean (SD)**					
Age (y)	58.9 (8.9)	60.5 (8.9)	61.9 (8.6)	62.2 (8.1)	<0.0001
BMI (kg/m^2^)	25.7 (4.2)	25.9 (4.1)	26.3 (4.4)	26.8 (4.1)	<0.0001
Systolic blood pressure (mmHg)	130.1 (17.3)	133 (19.4)	135.7 (18)	139.6 (19.2)	<0.0001
Total cholesterol (mmol/l)	5.58 (0.90)	6.19 (1.00)	6.60 (1.13)	7.12 (1.27)	<0.0001
LDL-cholesterol (mmol/l)	3.6 (0.86)	3.97 (1.01)	4.25 (1.07)	4.5 (1.16)	<0.0001
HDL-Cholesterol (mmol/)	1.43 (0.35)	1.57 (0.41)	1.58 (0.41)	1.6 (0.45)	<0.0001
Triglycerides (mmol/l)	1.21 (0.55)	1.45 (0.68)	1.74 (0.9)	2.33 (1.48)	<0.0001
Plasma vitamin C (µmol/l)	57.6 (20.2)	58.4 (20.2)	58.8 (19.6)	58 (20.7)	0.65
Alcohol (units/wk)	3.7 (4.6)	4.1 (5.0)	4.3 (5.7)	4.4 (6.2)	0.06
Plasma total PFA (µmol/l)	3,522.2 (408.9)	4,286.5 (327.6)	4,983.1 (447.8)	6,483.5 (1035.4)	<0.0001
Saturated PFA (µmol/l)	1,390.1 (146.7)	1,707.7 (75.6)	1,998.8 (99.1)	2,599.2 (369.4)	<0.0001
Odd chain PFA (µmol/l)	23.1 (5.1)	27.4 (5.3)	31.3 (5.9)	38.2 (8.7)	<0.0001
Omega 6 PFA (µmol/l)	1,434.9 (229.6)	1,719.2 (238.3)	1,959.5 (295.7)	2,509.6 (510.2)	<0.0001
Omega 3 PFA (µmol/l)	277.5 (92.1)	340.2 (113.3)	404.5 (144.2)	526.4 (186.5)	<0.0001
Monounsaturated PFA (µmol/l)	396.5 (69.5)	492 (88.1)	589 (113.6)	810.1 (208.9)	<0.0001
Trans PFA (µmol/l)	5.6 (3.3)	6.7 (3.3)	7.5 (3.8)	9.4 (4.7)	<0.0001
**Percent (** ***n*** **)**					
History of diabetes	1.4 (646)	2.2 (847)	2.8 (904)	2.6 (1,043)	0.27
Current smoker	9.8 (64)	9.4 (81)	8.1 (75)	11.4 (121)	0.1
Physically inactive	27.5 (180)	30.1 (260)	29.5 (274)	32.7 (350)	0.33
No educational qualifications	44.6 (292)	47.7 (413)	55.3 (514)	55.6 (595)	<0.0001
Manual social class	41.7(268)	38.2 (324)	33.9 (306)	36.7 (381)	0.02

*: *p*-Value using analysis of covariance.

Table 3 shows odds ratios for CHD by quartile of plasma phospholipid FA concentration using different multivariate models. The final column shows the odds ratio for CHD using FA concentrations as continuous variables, per approximate SD increase. Table 4 shows similar analyses using FA mol% quartiles.

**Table 3:** Odds ratios for CHD in men and women, EPIC-Norfolk 1993–2009 by quartile of plasma PFA concentration, age and sex adjusted, and multivariate adjusted and as a continuous variable, per approximate SD increase.

Plasma Phospholipid Fatty Acid	Categorical Odds Ratio (95% CI) per Quartile of PFA µmol/l				Continuous Odds Ratio (95% CI) per SD FA Increase	*p*-Value
	1	2	3	4		
**Total PFAs**						
Age and sex adjusted	1	1.06 (0.92–1.23)	1.06 (0.92–1.23)	1.11 (0.96–1.30)	1.05 (0.99–1.11)	0.10
Age, sex, PFA, and covariates adjusted^a^	1	1.08 (0.92–1.26)	1.07 (0.92–1.25)	1.06 (0.90–1.25)	1.02 (0.96–1.08)	0.60
Age, sex, PFA, and covariates adjusted^b^	1	0.97 (0.83–1.15)	0.90 (0.76–1.07)	0.84 (0.70–1.00)	0.92 (0.87–0.99)	0.02
**Saturated PFAs**						
Age and sex adjusted	1	1.00 (0.86–1.16)	1.06 (0.92–1.23)	1.19 (1.03–1.39)	1.09 (1.03–1.14)	0.002
Age, sex, and other PFA adjusted^c^	1	1.16 (0.97–1.40)	1.42 (1.12–1.79)	1.83 (1.37–2.46)	1.42 (1.23–1.61)	<0.0001
Age, sex, PFA, and covariates adjusted^a^	1	1.24 (1.01–1.51)	1.47 (1.14–1.90)	1.75 (1.27–2.41)	1.37 (1.19–1.50)	<0.0001
Age, sex, PFA, and covariates adjusted^b^	1	1.15 (0.94–1.41)	1.27 (0.98–1.64)	1.36 (0.98–1.89)	1.15 (0.99–1.33)	0.07
**Odd-chain PFAs**						
Age and sex adjusted	1	0.88 (0.74–0.99)	0.79 (0.68–0.92)	0.84 (0.72–0.98	0.97 (0.95–1.00)	0.05
Age, sex, and other PFA adjusted^c^	1	0.81 (0.69–0.95)	0.69 (0.58–0.82)	0.67 (0.54–0.80)	0.94 (0.91–0.98)	0.004
Age, sex, PFA, and covariates adjusted^a^	1	0.83 (0.70–0.98)	0.74 (0.62–0.90)	0.73 (0.59–0.91)	0.94 (0. 90–0.97)	0.001
Age, sex, PFA, and covariates adjusted^b^	1	0.81 (0.68–0.95)	0.71 (0.59–0.86)	0.67 (0.54–0.84)	0.93 (0.89–0.97)	<0.001
**Omega-6 polyunsaturated PFAs**						
Age and sex adjusted	1	0.99 (0.85–1.15)	0.97 (0.94–1.13)	1.02 (0.77–1.19)	1.01 (0.95–1.06)	0.73
Age, sex, and other PFA adjusted^c^	1	0.91 (0.76–1.07)	0.81 (0.66–0.99)	0.79 (0.62–0.99)	0.84 (0.76–0.92)	<0.0001
Age, sex, PFA, and covariates adjusted^a^	1	0.93 (0.77–1.11)	0.82 (0.66–1.01)	0.77 (0.60–0.99)	0.83 (0.75–0.92)	0.001
Age, sex, PFA, and covariates adjusted^b^	1	0.89 (0.74–1.07)	0.79 (0.64–0.98)	0.75 (0.59–0.97)	0.85 (0.77–0.95)	0.003
**Omega-3 polyunsaturated PFAs**						
Age and sex adjusted	1	1.03 (0.89–1.20)	1.00 (0.96–1.16)	0.94 (0.81–1.09)	0.96 (0.90–1.02)	0.19
Age, sex, and other PFA adjusted^c^	1	1.02 (0.87–1.19)	0.93 (0.78–1.10)	0.85 (0.70–1.02)	0.89 (0.82–0.97)	0.006
Age, sex, PFA, and covariates adjusted^a^	1	1.08 (0.92–1.28)	1.09 (0.90–1.31)	1.10 (0.90–1.36)	1.01 (0.92–1.11)	0.82
Age, sex, PFA, and covariates adjusted^b^	1	1.05 (0.89–1.25)	1.04 (0.87–1.26)	1.07 (0.87–1.32)	1.02 (0.93–1.12)	0.65
**Monounsaturated PFAs**						
Age and sex adjusted	1	1.05 (0.92–1.22)	1.12 (0.97–1.30)	1.15 (0.99–1.34)	1.08 (1.03–1.13)	0.003
Age, sex, and other PFA adjusted^c^	1	1.10 (0.93–1.30)	1.17 (0.96–1.43)	1.17 (0.92–1.48)	1.10 (1.01–1.20)	0.04
Age, sex, PFA, and covariates adjusted^a^	1	0.96 (0.80–1.16)	0.96 (0.78–1.19)	0.89 (0.69–1.16)	0.99 (0.90–1.09)	0.86
Age, sex, PFA, and covariates adjusted^b^	1	0.97 (0.81–1.17)	1.01 (0.81–1.25)	0.97 (0.74–1.37)	1.04 (0.95–1.15)	0.39
**Trans PFAs**						
Age and sex adjusted	1	0.91 (0.77–1.08)	0.95 (0.81–1.13)	1.01 (0.86–1.20)	1.01 (0.96–1.07)	0.82
Age, sex, and other PFA adjusted^c^	1	0.95 (0.79–1.11)	0.97 (0.81–1.15)	1.00 (0.84–1.20)	1.00 (0.94–1.07)	0.94
Age, sex, PFA, and covariates adjusted^a^	1	0.90 (0.75–1.08)	0.92 (0.76–1.11)	0.93 (0.76–1.13)	0.99 (0.92–1.05)	0.66
Age, sex, PFA, and covariates adjusted^b^	1	0.88 (0.73–1.06)	0.90 (0.74–1.09)	0.89 (0.73–1.09)	0.98 (0.91–1.05)	0.50

*p*-Values derived from Wald statistic.

a BMI, smoking, alcohol intake, physical activity, plasma vitamin C, social class, education, diabetes, systolic blood pressure.

b BMI, smoking, alcohol intake, physical activity, plasma vitamin C, social class, education, diabetes, systolic blood pressure, and cholesterol.

c PFAs grouped in categories.

**Table 4:** Odds ratios for CHD in men and women, EPIC-Norfolk 1993–2009 by quartile of plasma PFA mol%, age and sex adjusted, and multivariate adjusted and as a continuous variable, per approximate standard deviation increase.

Plasma Phospholipid Fatty Acid	Categorical Odds Ratio (95% CI) per Quartile of Mol% PFA				Continuous Odds Ratio (95% CI) per SD % FA Increase	*p*-Value
	1	2	3	4		
**Saturated even chain PFAs**						
Age and sex adjusted	1	1.10 (0.95–1.28)	1.22 (1.05–1.42)	1.34 (1.16–1.56)	1.18 (1.10–1.28)	<0.0001
Age, sex, and covariates adjusted^a^	1	1.13 (0.96–1.32)	1.21 (1.04–1.42)	1.25 (1.7–1.46.)	1.14 (1.05–1.24)	0.002
Age, sex, covariates, and cholesterol adjusted^b^	1	1.11 (0.94–1.30)	1.19 (1.01–1.39)	1.21 (1.03–1.42)	1.11 (1.02–1.21)	0.01
**Odd-chain PFAs**						
Age and sex adjusted	1	0.85 (0.73–0.98)	0.74 (0.64–0.87)	0.77 (0.66–0.89)	0.89 (0.84–0.94)	<0.0001
Age, sex, and covariates adjusted^a^	1	0.90 (0.76–1.05)	0.84 (0.72–0.99)	0.87 (0.74–1.03)	0.93 (0.88–0.99)	0.02
Age, sex, covariates, and cholesterol adjusted^b^	1	0.91 (0.77–1.07)	0.87 (0.74–1.03)	0.93 (0.79–1.10)	0.93 (0.88–0.99)	0.02
**Omega-6 Polyunsaturated PFAs**						
Age and sex adjusted	1	0.92 (0.79–1.06)	0.82 (0.71–0.96)	0.82 (0.71–0.95)	0.90 (0.85–0.95)	<0.0001
Age, sex, and covariates adjusted^a^	1	0.92 (0.79–1.08)	0.84 (0.72–0.98)	0.95 (0.72–0.99)	0.90 (0.85–0.96)	0.001
Age, sex, covariates, and cholesterol adjusted^b^	1	0.92 (0.79–1.08)	0.85 (0.73–1.00)	0.86 (0.73–1.00)	0.92 (0.87–0.98)	0.01
**Omega-3 polyunsaturated PFAs**						
Age and sex adjusted	1	1.02 (0.88–1.18)	0.83 (0.72–0.97)	0.84 (0.73–0.98)	0.92 (0.86–0.98)	0.01
Age, sex, and covariates adjusted^a^	1	1.07 (0.92–1.25)	0.89 (0.76–1.05)	1.00 (0.85–1.17)	1.00 (0.93–1.08)	0.97
Age, sex, covariates, and cholesterol adjusted^b^	1	1.10 (0.94–1.30)	0.90 (0.77–1.05)	0.97 (0.84–1.26)	1.00 (0.93–1.07)	0.98
**Monounsaturated PFAs**						
Age and sex adjusted	1	0.91 (0.78–1.05)	0.99 (0.85–1.14)	1.20 (1.04–1.39)	1.10 (1.04–1.15)	<0.001
Age, sex, and covariates adjusted^a^	1	0.90 (0.77–1.05)	0.92 (0.79–1.08)	1.03 (0.88–1.20)	1.04 (0.99–1.11)	0.16
Age, sex, covariates, and cholesterol adjusted^b^	1	0.93 (0.80–1.09)	0.95 (0.81–1.11)	1.06 (0.90–1.24)	1.03 (0.98–1.09)	0.24
**Trans PFAs**						
Age and sex adjusted	1	0.91 (0.77–1.08)	0.95 (0.81–1.12)	1.01 (0.86–1.20)	1.01 (0.96–1.07)	0.82
Age, sex, and covariates adjusted^a^	1	0.84 (0.70–1.00)	0.90 (0.75–1.07)	0.95 (0.79–1.13)	1.00 (0.94–1.06)	0.99
Age, sex, covariates, and cholesterol adjusted^b^	1	0.84 (0.71–1.01)	0.90 (0.75–1.08)	0.96 (0.80–1.15)	1.00 (0.94–1.06)	0.98

*p*-Value derived from Wald statistic. PFAs grouped according to quartiles of mo% total FAs.

a BMI, smoking, alcohol intake, physical activity, plasma vitamin C, social class, education, diabetes, systolic blood pressure.

b BMI, smoking, alcohol intake, physical activity, plasma vitamin C, social class, education, diabetes, systolic blood pressure, and cholesterol.

There was no overall significant relationship between total plasma phospholipid FA concentration and CHD. The weak but significantly positive association of CHD with plasma phospholipid SFA concentration increased in strength and significance after adjusting for other FA concentrations, with an odds ratio of 1.83 (95% CI 1.37–2.46, *p<*0.0001) in Q4 versus Q1 and a gradient of increasing risk throughout the distribution. This association hardly changed after multivariate adjustment apart from substantial attenuation after adjustment for cholesterol indicating much of the association between SFA and CHD was likely to be mediated through blood cholesterol levels.

In contrast, plasma phospholipid odd chain FA concentrations were inversely associated with lower CHD risk in all models. The odds ratio for Q4 versus Q1 was 0.67 (95% CI 0.54–0.80).

Plasma phospholipid n-6 PUFA concentrations were not significantly related to risk of CHD in the simple age- and sex-adjusted models, but after adjusting for other FAs, the relationship was significantly inverse. The odds ratio for CHD for Q4 versus Q1 was 0.84 (95% CI 0.76–0.92, *p<*0.0001).

Plasma phospholipid n-3 PUFA concentrations inversely related to CHD after adjusting for other FAs, but this was no longer significant after adjusting for other covariates. Plasma phospholipid MUFA concentrations showed a positive association with CHD before and after adjusting for other FAs though this relationship did not remain significant after adjusting for other covariates. Plasma phospholipid trans-fatty acid concentrations were not significantly associated with CHD.

Analyses using FA mol% rather than concentrations (Table 4) showed similar results with plasma phospholipid SFA positively and n-6 PUFA inversely significantly related to CHD.

Relationships were consistent in men and women in sex-stratified analyses (unpublished data) and also after further adjustment for dietary total energy, protein, carbohydrate, and fibre assessed using food frequency questionnaires (Text S2).

Table 5 shows mean plasma concentrations for individual phospholipid FAs.

**Table 5:** Mean plasma concentrations of individual PFAs in men and women, EPIC-Norfolk and age- and sex-adjusted odds ratios for CHD.

PFA	Plasma Concentration µmol/l (SD)	mol%	Odds Ratios CHD (95% CI) Age, Sex, and PFA Adjusted^a^	*p*-Value	Odds Ratios CHD (95% CI) Multivariate Adjusted^b^	*p*-Value
Total PFAs	4,768.4(1266)	100	1.05 (0.99–1.11)	0.10	1.02 (0.96–1.08)	0.60
**Saturated even chain PFA**	**1,905.0 (492.4)**	**39.9**	**1.42 (1.23–1.61)**	**<0.0001**	**1.37 (1.19–1.58)**	**<0.0001**
14:0 myristic	17.6 (10.6)		0.98 (0.89–1.09)	0.71	0.97 (0.87–1.08)	0.97
16:0 palmitic	1,256.8 (332.3)		1.24 (1.07–1.45)	0.006	1.37 (1.16–1.62)	<0.001
18:0 stearic	628.0 (184.0)		1.68 (1.38–2.05)	<0.0001	1.56 (1.25–1.93)	<0.0001
**Odd chain fatty PFA**	**29.1(8.5)**	**0.6%**	**0.94 (0.91–0.98)**	**0.004**	**0.94 (0.90–0.97)**	**0.001**
15:0 pentadecanoic	9.2(3.1)		0.95 (0.86–1.04)	0.28	0.98 (0.89–1.09)	0.79
17:0 heptadecanoic/margaric	18.4 (5.9)		0.91 (0.83–1.01)	0.07	0.91 (0.82–1.01)	0.07
**Omega-6 polyunsaturated PFA**	**1,888.9(492.4)**	**39.6%**	**0.84 (0.76–0.92)**	<0.0001	**0.83 (0.75–0.92)**	**0.001**
18:2n-6 linoleic	1,166.5(330.0)		0.66 (0.58–0.75)	<0.0001	0.70 (0.61–0.81)	<0.0001
18:3n-6 gamma-linolenic	6.0 (5.2)		1.05 (0.98–1.13)	0.18	1.05 (0.97–1.14)	0.21
20:2n-6 eicosadienoic	20.5(8.8)		1.06 (0.95–1.18)	0.33	1.09 (0.97–1.23)	0.14
20:3n-6 dihomo-gamma-linolenic	194.6(83.6)		1.22 (1.08–1.39)	0.002	1.06 (0.92–1.22)	0.44
20:4n-6 arachidonic	475.2(163.9)		0.86 (0.76–0.97)	0.018	0.84 (0.74–0.97)	0.013
22:4n-6 docosatetrahexanoic	16.6 (9.9)		1.10 (1.00–1.20)	0.04	1.08 (0.98–1.18)	0.11
22:5n-6 docosapentenoic	9.7(5.8)		0.87 (0.78–0.96)	0.007	0.87 (0.77–0.97)	0.016
**Omega-3 polyunsaturated PFA**	**377.0(165.7)**	**7.9%**	**0.89 (0.82–0.97)**	**0.006**	**1.01 (0.92–1.11)**	**0.82**
18:3n-3 alpha-linolenic	11.4(6.4)		0.97 (0.88–1.07)	0.58	0.98 (0.89–1.09)	0.70
20:5n-3 eicosapentanoic	63.1(45.2)		1.01 (0.92–1.12)	0.83	1.00 (0.89–1.11)	0.95
22:5n-3 docosapentaenoic	65.1(28.0)		0.72 (0.63–0.84)	<0.0001	0.84 (0.72–0.98)	0.03
22:6n-3 docosahexanoic	237.4 (106.2)		0.95 (0.84–1.07)	0.39	0.96 (0.85–1.09)	0.56
**Monounsaturated PFA**	**568.4 (213.5)**	**11.9%**	**1.10 (1.01–1.20)**	**0.04**	**0.99 (0.90–1.09)**	**0.86**
16:1n-7 palmitoleic	38.4 (19.5)		0.98 (0.84–1.14)	0.76	0.91 (0.77–1.07)	0.26
18:1n-7 cis cis-vaccenic	45.9 (17.5)		1.10 (1.00–1.22)	0.06	1.17 (1.06–1.31)	0.003
18:1n-9 cis cis-oleic	474.0 (184.9)		1.01 (0.85–1.20)	0.90	0.91 (0.75–1.09)	0.31
20:1n-9 gadoleic	7.6 (6.1)		1.18 (1.04–1.33)	0.008	1.20(1.05–1.36)	0.006
**Trans PFA**	**7.2 (4.1)**	**0.2%**	**1.00 (0.94–1.07)**	**0.94**	**0.99 (0.92–1.05)**	0.66
16:1n9 trans palmitelaidic	2.4 (1.5)		0.97 (0.90–1.05)	0.48	0.93 (0.85–1.01)	0.08
18:1n9 trans elaidic	4.8 (3.5)		1.01 (0.93–1.09)	0.88	0.99 (0.91–1.07)	0.74

a Adjusted for age, sex, and all individual PFAs in the same model for individual PFAs, families of PFA for the families, all as continuous variables.

b Adjusted for age, sex, other PFA, BMI, smoking, physical activity, alcohol intake, social class, education, blood pressure.

Table 5 also shows odds ratios for CHD for each individual FA, adjusting for age and sex and the other individual FAs listed, as continuous variables and then with multivariate adjustment excluding cholesterol. Odds ratios are shown per approximate SD increase in plasma concentration of the relevant phospholipid FA.

Stearic acid (18:0) concentration was most strongly positively related to CHD (OR 1.68, 1.38–2.05, *p<*0.0001) followed by palmitic acid (16:0) concentration (OR 1.24, 1.07–1.45, *p* = 0.006). There was no observed association with myristic acid (14:0) concentration (OR 0.98, 0.89–1.09).

Individual plasma phospholipid n-6 PUFA concentrations showed marked heterogeneity in the relationship with CHD. Linoleic acid (18:2n-6) was most strongly inversely related, OR 0.66 (0.58–0.75, *p<*0.001). Inverse associations were also observed for AA (20:4n-6), OR 0.86 (0.76–0.97, *p = *0.02), and docosapentenoic acid (22:5n-6) OR 0.87 (0.78–0.96, *p = *0.007). However, di-homo-gamma-linolenic acid (DGLA) (20:3n-6) positively related to CHD OR 1.22 (1.08–1.32, *p = *0.002).

Of the n-3 PUFAs, only plasma phospholipid docosapentaenoic acid (22:5n-3) concentration was significantly inversely associated with CHD OR 0.72 (0.63 01500.84, *p<*0.00001). The main monounsaturated FA, cis-oleic was not related to CHD (OR 1.01, 0.85–1.20). Plasma phospholipid concentration of gadoleic acid (20:1n-9) was significantly positively related to CHD (OR 1.18,1.04–1.33, *p = *0.008).

Plasma phospholipid concentrations of the two most common trans-fatty acids measured: palmitelaidic (16:1n9 trans) and elaidic (18:1n9 trans) were not related to CHD at these very low concentrations.

## Discussion

There was no overall association between total plasma phospholipid FA concentration and CHD and only weak associations were observed for each family of FAs considered in isolation. However, when other FAs were taken into account, plasma phospholipid SFA concentrations strongly positively and n-6 PUFA concentrations inversely related to subsequent risk of incident CHD. For plasma phospholipid SFA concentrations, those in Q4 versus Q1 had approximately 75% higher CHD risk, and for n-6 PUFA concentrations, 25% lower risk in Q4 versus Q1. In contrast, plasma odd chain phospholipid FA concentrations were inversely related to CHD with a lower risk of about 30% in Q4 versus Q1. These relationships were independent of potential confounders including age, BMI, smoking, alcohol intake, physical activity, plasma vitamin C, history of diabetes, social class, education, as well as systolic blood pressure. The relationship between plasma phospholipid SFA concentration and CHD was attenuated after adjusting for blood cholesterol concentrations, indicating that at least some of the relationship could be mediated through lipid concentrations. Plasma phospholipid N-3 PUFA concentrations inversely related to CHD after adjusting for other FAs, but this relationship was no longer apparent after multivariate adjustment. There were no overall relationships with plasma phospholipid MUFA and trans-fatty acid concentrations. Results were consistent using FA mol%.

Observational and intervention studies indicate that blood FA profiles may be biomarkers for dietary FA intake though dietary associations are stronger for the FAs such as linoleic acid and alpha linolenic acid intake, which humans cannot produce [8],[10],[13].

In this study, a deuterium-labelled phospholipid internal standard allowed accurate determination of individual phospholipid FA plasma concentrations. Previous prospective studies examining individual FAs and CHD used percent composition of FAs in plasma rather than plasma concentration and not all measured the full range of FAs necessary to determine correctly the denominator [14],[15],[21].

Nevertheless, several such studies reported positive relationships between plasma phospholipid SFA composition and CHD, inverse relationships between plasma phospholipid PUFA composition, and no overall relationship with MUFA composition, consistent with our findings [14],[19],[21]. The consistent positive associations of SFA in cohorts using biomarker FA profiling is in marked contrast to reports using dietary assessments of saturated fat intake and CHD [2].

The lack of association between dietary saturated fat intake and CHD in population studies is unsurprising given the large measurement errors in dietary assessment of fat intake through recall errors, ubiquity of fats leading to quantification difficulties, and critically, huge variability in FA composition of foods such that discrimination between different fats is problematic.

Different types of fat using the conventional groupings of SFA, n-6 and n-3 PUFA, MUFA and trans-fatty acid, have varying biological and health effects. Though it is generally believed that saturated and transfats are adversely and unsaturated fats beneficially related to CHD risk [1],[7], the balance between the different fats may be more important than any single group alone. Keys noted the saturated/unsaturated fat ratio was critical for predicting cholesterol levels and CHD [29]. In the current study, we found no overall relationship between total FAs and CHD. Trials substituting unsaturated fat for saturated fat, altering the ratios have reported more consistently reduction in cardiovascular disease [6],[7], indicating that the balance between different fats is crucial.

Additionally even within these families, increasing evidence indicates individual FAs have different metabolic and health effects. Such heterogeneity in the relationship between individual SFAs or n-6 PUFAs and CHD noted in several prospective studies may explain variable associations of total SFAs and n-6 PUFA with CHD in different populations depending on the distribution of individual FAs.

In this study, stearic acid (18:0) plasma phospholipid concentration was the SFA most strongly positively related to CHD, followed by palmitic acid (16:0) concentration. Myristic acid (14:0) was not related to CHD risk. Of the n-3 PUFAs, only docosapentaenoic acid, (22:5n-3) plasma phospholipid concentration inversely related to CHD. This is not surprising as n-3PUFAs have primarily been associated with arrhythmic cardiac death [30].

Of the n-6PUFAs, the plasma phospholipid concentration of linoleic acid (18:2n-6) and AA (20:4n-6) inversely related to CHD, but DGLA (20:3n-6) positively related to CHD.

The n-6 PUFA AA (22:4n6) is the substrate for series 2 thromboxanes and prostanoids metabolites believed to be proinflammatory and thrombotic, in contrast to DGLA (22:3n6) for which the eicosanoid metabolites, series 1 thromboxanes and prostanoids are thought to be anti-inflammatory and antithrombotic [31]. DGLA is therefore believed to be beneficial for CHD in contrast to AA. However, we observed the converse, a positive relationship between DGLA and negative relationship between AA and CHD, unexpected findings also noted by other prospective studies [14],[18],[19],[21], including the ARIC study (282 CHD events) [21], a nested case control study within the Multiple Risk Factor Intervention Trial (94 CHD cases, 94 controls) [19], a Swedish cohort (153 CHD events) [18], and the Whitehall study (116 events) [14]. The Swedish investigators hypothesised their observed inverse relationship between AA/DGLA ratio and CHD reflected activity of delta5-desaturase enzyme [18].

Results for odd chain FAs (15:0; 17:0) require comment. Though often included with SFAs, they are metabolised differently from even chain SFAs. The 15:0 and 17:0 FAs are ruminant specific and suggested to be biomarkers of milk or dairy intake [12]. Studies reporting on odd chain FAs have inconsistent results. The Nurses Health Study (166 cases, 327 controls) reported an odds ratio of 2.36 for CHD between top and bottom tertiles of odd chain plasma FA composition [20]. In contrast, a Swedish cohort (446 cases, 558 controls) reported a standardized OR for CHD of 0.76 in women and 0.91 in men [23]. We found an odds ratio of 0.67 in Q4 versus Q1 for odd chain FAs. Despite the contribution of dairy produce to saturated FA composition of the diet, a recent reappraisal concluded there is no consistent evidence that dairy food consumption is associated with a higher risk of cardiovascular disease [32]. Milk or dairy consumption has been reported to be associated with lower risk of metabolic syndrome [33], heart disease [34], and hypertension [35], and the DASH trial reported lower blood pressure with a dietary intervention including low fat milk [36]. These associations need further exploration and confirmation.

The study has limitations. Although we conducted a large number of statistical tests, the analyses for the main FA families were designed using a priori hypotheses. Despite not being in line with the putative biological effects of their metabolites, the AA and DGLA results are consistent with previous studies in different settings in the US and Europe. The results for odd chain FAs have some previous support, but will need confirmation in future studies.

We only measured the two most common trans-fatty acids (16:1n9trans, 18:1n9trans). Though we were unable to assess the 18:2 transisomers associated with CHD in other studies [37], at the very low concentrations in a range more typical of European populations [11], there was little power to assess the trans-fatty acid–CHD relationship. We controlled for the major lifestyle and demographic factors related to CHD risk but cannot exclude residual confounding with other unknown factors.

We used only plasma FAs measured one point in time to characterise individuals. There is likely to be large intraindividual variation in FAs but such random measurement error is likely to attenuate any relationships, rather than produce spurious associations.

Future studies examining the relationship between FAs and CHD need to consider heterogeneity in the biological and health effects of individual FAs as well as the overall FA profile and balance between FAs. The ability to assess these associations in human populations has been constrained by the limitations of assessing dietary fat intake. Biochemical measures offer more objective and specific biomarkers, which may provide greater insight into exogenous dietary factors as well as endogenous metabolic processes which influence risk of chronic diseases.

These results indicate that high plasma phospholipid SFA and low PUFA (predominantly n-6 FAs) are associated with increased CHD risk but neither can be considered in isolation. It is beyond the remit of the current study to quantify in absolute amounts the relationship between blood FA concentrations and dietary intake of foods and nutrients, It is not clear how far the associations reflect complex interactions between dietary fat intake per se and FA metabolism, which may have both genetic and other exogenous influences. Nevertheless, the major sources of dietary n-6 PUFA, i.e., linoleic acid in most western populations, are vegetable oils such as sunflower oil, corn oil, soybean oil, and canola oil, and nuts and seeds.

While dietary recommendations should focus on general patterns of food intake rather than individual nutrients [38], recommendations still need to be based on knowledge of the biological roles of different nutrients and balance between different nutrients in foods. Just as recommendations now acknowledge differences in health effects of different families of fats, so they should evolve to reflect increasingly discriminating understanding of individual FA metabolism, and their interactions. Early guidelines to prevent CHD recommended reductions in saturated fat but little consistency as to what might be substituted: other fats, protein, or carbohydrate. Our results add to the accumulating evidence that substitution of saturated fat by n-6 polyunsaturated fat may have more CHD benefits [33],[39],[40].

## Supporting Information

Text S1 **Detailed description of the analytic methods for plasma phospholipid measurements.**
(DOC)

Text S2 **Odds ratio for plasma PFAs per approximate SD, PFA increase, adjusted for age, sex, BMI, smoking, alcohol intake, physical activity, plasma vitamin C, social class, education, diabetes, systolic blood pressure, total energy intake, total carbohydrate intake, total protein intake. and fibre intake.**
(DOC)

## Abbreviations

AA: arachidonic acid
BMI: body mass index
CHD: coronary heart disease
DGLA: di-homo-gamma-linolenic acid
EPIC: European Prospective Investigation into Cancer
FA: fatty acid
HDL: high-density lipoprotein
LDL: low-density lipoprotein
MUFA: mono-unsaturated fatty acid
PUFA: polyunsaturated fatty acid
SD: standard deviation
SFA: saturated fatty acid
