# Supplementary material for: Plasma Phospholipid Fatty Acid Concentration and Incident Coronary Heart Disease in Men and Women: The EPIC-Norfolk Prospective Study
Source: PLoS Med. 2012 Jul 3;9(7):e1001255. doi: 10.1371/journal.pmed.1001255 (PMC3389034; doi:10.1371/journal.pmed.1001255)
Supplement: Text S1 — Detailed description of the analytic methods for plasma phospholipid measurements. (DOC) [file pmed.1001255.s001.doc]

**Text S1 Detailed description of the analytic methods for plasma phospholipid measurements**.

Citrated plasma straws were retrieved from liquid nitrogen storage, thawed at room temperature and 20 μg of di-palmitoyl-D_31_-phosphatidylcholine (Sigma, St. Louis, MO) internal standard was added to each 200 μl plasma sample. Following extraction of total lipids with chloroform/methanol, phospholipids were further purified by adsorption chromatography (LC-Si SPE, Supelco/Sigma, St. Louis, MO), transmethylated to fatty acid methyl esters and extracted with hexane. Analysis was carried out by gas chromatography with flame ionisation detection (220°C) using a 30 m x 0.32 mm x 0.2 um SP2340 fused silica capillary column (Supelco/Sigma, St. Louis, MO). Carrier gas was Helium at a constant flow of 1.3 ml/min. Samples of 0.5 µl were introduced onto the column via on-column injection. The column was held initially for 1 min at 65°C, then programmed at 5°C/min to 135°C, then at 2°C/min to 200°C, and finally at 10°C/min to 220°C. Run time was 60 min.

Identification of individual fatty acid methyl esters was based on comparison with retention times of authentic standards (Sigma, St. Louis, MO). Plasma concentrations were measured by comparison of peak areas of individual fatty acids with the peak area of the palmitoyl-D_31_-fatty acid methyl ester internal standard using individual calibration curves for each of the 22 fatty acid methyl esters measured. The chromatographic peak for palmitoyl-D_31_-fatty acid methyl ester elutes about 1 minute earlier than non-labelled palmitoyl fatty acid methyl ester in a zone free of interference from other peaks. Each chromatogram was integrated automatically and checked for accuracy and specificity by a laboratory technician. The analytical method allowed for the analysis of 22 individual fatty acids with chain lengths between 14 and 22 carbons belonging to 6 classes of fatty acids: saturated even chain, SFA (14:0, 16:0, 18:0); odd chain FA(15:0, 17:0); Omega-6 polyunsaturated, n-6 PUFA (18:2n-6, 18:3n-6, 20:2n-6, 20:3n-6, 20:4n-6, 22:4n-6, and 22:5n-6); Omega-3 polyunsaturated, n-3 PUFA (18:3n-3, 20:5n-3, 22:5n-3, 22:6n-3); Mono-unsaturated, MUFA, (16:1n-7, 18:1n-7cis, 18:1n-9cis, 20:1n-9) and transFA (16:1n9trans, 18:1n9trans). Results were reported as µmolar plasma concentrations and mol% for each individual FA, taking into account the large difference in molecular mass between the shortest (myristic acid, 14:0) and longest (docosapentaenoic acid, 22:5n-3) chain FA measured. Analytical quality control was carried out by the daily use of standard quality control plasma samples. The CVs for the major fatty acids were between 3% to 13%.

Samples were identified only by a code number and cases and controls assorted within each analytic batch.
