# Supplementary material for: Plasma Phospholipid Fatty Acid Concentration and Incident Coronary Heart Disease in Men and Women: The EPIC-Norfolk Prospective Study
Source: PLoS Med. 2012 Jul 3;9(7):e1001255. doi: 10.1371/journal.pmed.1001255 (PMC3389034; doi:10.1371/journal.pmed.1001255)
Supplement: Text S2 — Odds ratio for plasma PFAs per approximate SD, PFA increase, adjusted for age, sex, BMI, smoking, alcohol intake, physical activity, plasma vitamin C, social class, education, diabetes, systolic blood pressure, total energy intake, total carbohydrate intake, total protein intake. and fibre intake. (DOC) [file pmed.1001255.s002.doc]

**Text S2**

Odds ratio for plasma phospholipid fatty acids(PFA) per approximate standard deviation (s.d.) PFA increase, adjusted for age, sex, body mass index, smoking, alcohol intake, physical activity, plasma vitamin C, social class, education, diabetes, systolic blood pressure, total energy intake, total carbohydrate intake, total protein intake and fibre intake

_________________________________________________________________________________________________

OR (95% CI) per P value

s.d. PFA increase

__________________________________________________________________________________________________

**Total phospholipid fatty acids** 1.01 (0.95-1.08) 0.68

**Saturated even chain phospholipid fatty acids** 1.35 (1.17-1.56 ) <0.0001

**Odd-chain phospholipid fatty acids** 0.95 (0.91-0.98) 0.008

**Omega-6 Polyunsaturated phospholipid fatty acids** 0.84 (0.75-0.93) <0.001

**Omega-3 polyunsaturated phospholipid fatty acids** 1.00 (0.91-1.09) 0.95

**Monounsaturated phospholipid fatty acids** 1.00 (0.90-1.10) 0.92

**Trans phospholipid fatty acids** 0.99 (0.92-1.06) 0.67

__________________________________________________________________________________________________
